# Combined Treatment of Acidic Electrolyzed Water and High-Voltage Electrostatic Field Improves the Storage Quality of Huping Jujube (*Ziziphus jujuba* Mill. cv. Huping)

**DOI:** 10.3390/foods12142762

**Published:** 2023-07-20

**Authors:** Xiaojie Chang, Yueguang Liang, Tianjing Guo, Yu Wang, Jiali Yang

**Affiliations:** 1 College of Horticulture, Shanxi Agricultural University, Taigu 030800, China; b20211025@stu.sxau.edu.cn; 2 Life Sciences Department, Yuncheng University, Yuncheng 044000, China; 3 College of Food Science and Engineering, Shanxi Agricultural University, Taigu 030800, China; z20213474@stu.sxau.edu.cn (Y.L.); z20213509@stu.sxau.edu.cn (T.G.); jiayang@sxau.edu.cn (J.Y.)

**Keywords:** Huping jujube, acidic electrolyzed water, high-voltage electrostatic field, storage quality

## Abstract

Fresh jujube is prone to rapid deterioration after harvest due to its active metabolism and rich nutrients. This study aimed to investigate the effects of acidic electrolyzed water (AEW), a high-voltage electrostatic field (HVEF) and a combination of AEW and HVEF (AEW + HVEF) treatments on the storage quality of Huping jujube (*Ziziphus jujuba* Mill. cv. Huping) stored at 0 ± 1 °C for 90 days. The results showed that the fruits treated with AEW + HVEF exhibited better storage quality than those treated with either AEW or HVEF alone. Specifically, the fruits treated with AEW + HVEF maintained higher levels of nutrients and taste compounds, including total soluble solid (TSS), total soluble sugar, reducing sugar and titratable acidity (TA), as well as lower respiration rate, weight loss, decay index and TSS/TA ratio. Additionally, the AEW + HVEF treatment could delay the increase in reddening index, a* and color change (ΔE) values, and the decrease in L* and b* values, by retarding the degradation of chlorophyll and accumulation of carotenoids and flavonoids, thereby preserving the more desirable appearance color. Furthermore, the combined treatment could enhance the glutathione reductase (GR) activity and 2,2′-azino-bis-(3-ethylbenzothizoline)−6-sulfonic acid (ABTS) +-scavenging ability. Thus, the AEW + HVEF treatment is a potential method for Huping jujube preservation.

## 1. Introduction

Huping jujube (*Ziziphus jujuba* Mill. cv. Huping) is a special product of Taigu District, Jinzhong City, Shanxi Province, China. It is called Huping jujube because its fruit shape is small at the top, large at the bottom, slightly thin in the middle, and shaped like a pot or a bottle. It was identified as China’s National Geographic Indication product in 2007. Huping jujube is an excellent fresh-eating variety due to its thin peel, juicy taste, crisp texture and abundant bioactive substances [1,2]. However, Huping jujube (white mature stage) is usually harvested in mid-August when the average maximum temperature in Jinzhong City, the main producing area of Huping jujube, typically reaches around 30 °C. If stored at ambient temperature without any packaging or treatment, the jujubes will rapidly lose water and shrink within two or three days, as well as begin to turn red or even rot within a week, which seriously affects consumers’ willingness to purchase and damages the economic interests of growers [3,4]. Until now, many preservation methods have been applied to improve the storage quality of jujube, including glycine betaine [1], salicylic acid [5], propyl gallate [6], hydrogen sulfide [7], low temperature [8], ultraviolet [3], γ-irradiation [9] and X-ray irradiation [10]. Although these methods can preserve the quality to some extent, there are some inevitable limitations, such as high energy consumption, complex operation, safety and environmental pollution issues [1,3,5] Therefore, it is necessary to develop an environment-friendly, low-lost and high-efficiency treatment to improve the storage quality.

HVEF is a non-thermal, high-efficiency, low-cost physical preservation technology [11,12], which exhibits a uniform voltage distribution and superior stability than other discharge modes, including high-voltage direct current, alternate current and pulsed current [13]. In recent years, HVEF has been widely applied to improve the sensory quality, tissue structure and nutritional components of pakchoi [11], fresh-cut cabbage and baby corn [14], button mushroom [15], fresh-cut broccoli [16], persimmon [17], cherry tomato [18], tomato [19], cranberry [20], etc.

AEW can be produced on-site by the electrolysis of certain salt solutions (NaCl, MgCl_2_ and GaCl_2_) or hydrochloric acid solution in an electrolysis apparatus with a diaphragm [21,22,23]. AEW has three important physicochemical properties: lower pH values, higher oxidation-reduction potentials (ORPs) and a wide range of available chlorine concentrations (ACCs). The ACC can mainly be determined by Cl_2_, HOCl, O_2_, ^−^OCl and HCl, which are generated at the anode of electrolytic device [22,24]. Additionally, AEW can be converted to ordinary water when it comes into contact with organic matters or is diluted with tap water, posing no threat to the environment or human health [25]. Previous studies have showed that AEW can effectively kill various microorganisms and have positive effects on the storability and quality properties of blueberry [26], ‘Lingwu long’ jujube [27], longan [28,29], etc.

However, although AEW and HVEF treatments have been applied to improve the postharvest quality of fruits and vegetables, there have been no studies on the effects of single or combined treatment with AEW and HVEF on the storage quality of Huping jujube to the best of our knowledge. Therefore, this study aimed to investigate the effects of AEW, HVEF and AEW + HVEF treatments on the respiration rate, decay index, reddening index, nutrients and taste compounds, appearance color and antioxidant capacity of Huping jujube stored at 0 ± 1 °C for 90 days. Results were expected to provide a theoretical basis for further research on the regulation mechanism of senescence in Huping jujube.

## 2. Materials and Methods

### 2.1. HVEF Treatment System

**Figure 1:**
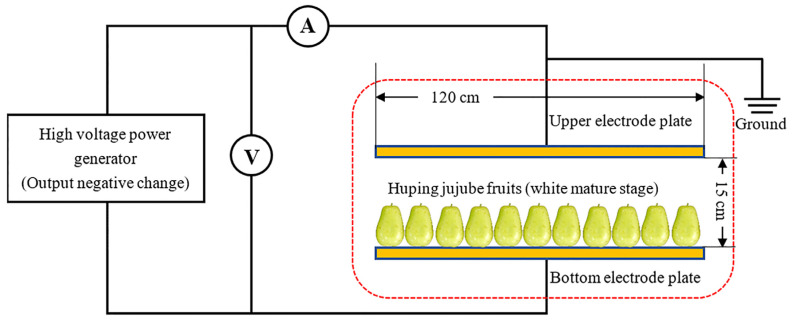
Schematic diagram of high voltage electrostatic field (HVEF) treatment.

The HVEF experimental system consisted of a high-voltage generator (DW-N303-1ACF 7, Dongwen High Voltage Power, Tianjin, China) with output of −30–0 kV, two parallel rectangular stainless electrode plates used as cathode or anode, one voltmeter and one ammeter. Based on the preliminary experiment results, the field intensity of −2 kV/cm was adopted. The jujubes were placed horizontally without overlapping. The schematic diagram of the HVEF system is shown in Figure 1.

### 2.2. AEW Preparation

AEW was produced by electrolyzing 1‰ NaCl solution in an electrolyzed water generator (XYS-C-12, Xin-yu Optical Electromechanical Co., Ltd., Baoji, China). Based on the preliminary experiment results, appropriate AEW treatment conditions were selected: pH of 2.8, ORP of 1550 ± 5 mV, ACC of 90 mg/L. The pH and ORP were determined using the pH and ORP meters (pH-208 and ORP986, Fu-an-pu-he Electronics Co., Ltd., Fuzhou, Fujian, China), respectively. The ACC of AEW was quantified by iodometry. 

### 2.3. Materials and Treatments

Huping jujubes were hand-harvested at the white mature stage (picking date: 17 August 2022.; maturity stage: 80 days after flowering) from an orchard in Xiaobai Township, Jinzhong City, Shanxi Province of China and transported to the Fruits and Vegetables Storage and Preservation Laboratory of Shanxi Agricultural University within 3 h. After being pre-cooled at 4 °C for 24 h, jujubes with uniform appearance and without flaws and injuries were selected, then stored at 0 ± 1 °C (relative humidity: 85–95%) for 90 days. The jujubes were randomly divided into five groups, with three biological replicates per group and 350 fruits per replicate. A total of 5250 jujubes were used in this study, with an average weight of 20 ± 2 g per jujube.

The groups were treated and tagged as follows: (1) CK group (untreated); (2) DW group (soaked in 15 L distilled water for 10 min, then air-dried for 2 h at room temperature); (3) AEW group (soaked in 15 L AEW for 10 min, then air-dried for 2 h at room temperature); (4) HVEF group (treated with −2 kV/cm HVEF for 3 h); and (5) AEW + HVEF group (soaked in 15 L AEW for 10 min and air-dried for 2 h, then treated with −2 kV/cm HVEF for 3 h). Then, the fruits were placed into perforated polyethylene bags (50 jujubes per bag) and stored at 0 ± 1 °C (relative humidity: 85–95%). The experimental cold storage used in this paper was built by Shanxi Jia-He-Zhong-Xing Refrigeration Equipment Co., Ltd. commissioned by Shanxi Agricultural University. The cold storage door and storage board are made of colored steel polyurethane material. The cold storage is equipped with a condenser (MCU-31NSJ, Panasonic Cold Chain Co., Ltd., Dalian, China), chiller (CC-CV6000H, Panasonic Cold Chain Co., Ltd., Dalian, China) and compressor (C-L22M8F, Panasonic Appliances Compressor Co., Ltd., Dalian, China). Ninety jujubes (30 jujubes × 3 replicates) were randomly chosen from each group at 15 d intervals for the analysis of physiochemical indicators, and the remaining sixty jujubes (20 jujubes × 3 replicates) were pitted, frozen by liquid nitrogen, and then placed at −80 °C for subsequent analysis. All measurements were performed in triplicate.

### 2.4. The Respiration Rate, Weight Loss, Decay Index and Reddening Index

About 500 g of jujubes per replicate were sealed in a 2.6 L LOCK&LOCK fresh-keeping box and placed in a storage environment (temperature: 0 ± 1 °C, relative humidity: 85–95%) for 4 h. Subsequently, the respiration rate was measured using a portable gas analyzer (F-940, Beijing Sunshine Yishida Technology Co., Ltd., Beijing, China). The result was expressed as mg·kg^−1^ h^−1^, according to the method of Yang et al. [5]

Fifty jujubes of each replicate were used to measure the decay index and reddening index based on the areas of decay and reddish peel, following the methods of Jia et al. [3] and Wang et al. [6], respectively.

Weight loss was determined using fifty fruits from each replicate and calculated following the method of Sang et al. [8].

### 2.5. TSS, TA, TSS/TA, Total Soluble Sugar and Reducing Sugar

TSS and TA were assessed using 5 g of pulp from 10 fruits in each replicate. TSS was quantified using a digital refractometer (PAL-1, Atago, Tokyo, Japan) following the method of Zhang et al. [1]. The results were recorded as a percentage (%). TA was measured as reported by Islam et al. [30] and stated as % of malic acid. The TSS/TA ratio was then calculated.

The procedures were as follows: Mix 3 g pulp from 10 fruits with a small amount of distilled water, then grind the mixture into a homogenate and transfer it to a beaker. Add 30 mL distilled water into the beaker, seal it with plastic film, boil it in boiling water for 30 min, take it out and filter the homogenate directly into a 100 mL volumetric flask after cooling. Recycle the residue into the beaker, add 30 mL distilled water and boil for extraction for another 10 min, then filter into the volumetric flask after cooling. Rinse the beaker and residue with distilled water, transfer them to the volumetric flask after filtration and adjust the volume to the scale. According to the sucrose standard curve, the total soluble sugar content was measured at 630 nm using the anthrone reagent method following the description of Jat et al. [9]. The results were expressed in %.

Mix 3 g pulp from 10 fruits with a small amount of distilled water in a mortar, grind the mixture into a homogenate and transfer it to a beaker. Add 30 mL distilled water into the beaker, seal it with plastic film and keep it in a water bath at 80 °C for 30 min so that the reducing sugar can be leached. After cooling, filter the extract and wash the residue with another 30 mL distilled water before filtering again. Collect both filtrates in a 100 mL volumetric flask and adjust the volume to the scale with distilled water. According to the glucose standard curve, the content of reducing sugar was measured using the 3,5-dinitrosalicylic acid method at 540 nm following the description of Lopes et al. [31]. The results were expressed in %.

### 2.6. The Contents of Chlorophyll, Carotenoids and Flavonoids

Quantities of 3 g of peels of 10 jujube fruits and 0.3 g sodium carbonate were mixed together, and immediately ground into homogenate with 80% acetone pre-cooled at −20 °C for 24 h in the dark. The homogenate was extracted at 4 °C under dark conditions for 30 min, and then centrifuged at 4 °C 12,000× *g* for 10 min. The residue was rinsed again with 20–30 mL 80% acetone until the residue had no green color. Finally, the volume of extraction was diluted to 50 mL with 80% acetone. The extraction was measured at 440 nm, 663 nm and 645 nm using a UV–Vis spectrophotometer (P4, Mepuda Instrument Co., Shanghai, China). The contents of chlorophyll and carotenoids were calculated according to the methods of Lv et al. [7] and Zhang et al. [11]. The results were expressed as mg·100 g^−1^.

Quantities of 3 g of peels of 10 jujube fruits were mixed with 15 mL of HCl-methanol solution (1%, *v/v*) pre-cooled at 4 °C. After grinding the mixture into a homogenate under ice bath conditions, the resulting homogenate was collected and diluted to a final volume of 30 mL. The extraction was carried out at 4 °C for 2 h, followed by centrifugation at 12,000× *g* for 20 min at the same temperature. The supernatant was then adjusted to a final volume of 30 mL with HCl-methanol solution (1%, *v/v*), and the OD value of the supernatant was measured at 325 nm. The results were stated as mg rutin equivalents (RE)·100 g^−1^ based on the standard curve with rutin according to the methods of Lv et at. [7] and Jiang et al. [32].

### 2.7. Color Characteristics

The skin color was measured with a colorimeter (NH310, Shenzhen ThreeNH Technology Co., Ltd., Shenzhen, China) at three points on the equator of 10 fruits, according to the method of Li et al. [33]. Data were recorded as L*, a* and b*. ΔE was calculated based on the method of Yang et al. [5]

### 2.8. The Glutathione Reductase (GR) Activity and 2,2′-Azino-Bis-(3-Ethylbenzothizoline)−6-Sulfonic Acid (ABTS) Radical Scavenging Ability

The procedures were as follows: Grind the mixture, which includes 10 g of frozen pulp tissue from 20 jujubes and 30 mL of 100 mmol/L, pH 7.5 phosphate buffer (containing 1 mmol/L EDTA) pre-cooled at 4 °C, into a homogenate under ice bath conditions. Then centrifuge the homogenate at 4 °C and 12,000× *g* for 30 min, and collect the supernatant to determine GR activity according to the method of Sang et al. [8]. One GR activity unit (U) was defined as a change of 0.01 per minute per gram of fresh weight at 340 nm using a UV–Vis spectrophotometer (P4, Mepuda Instrument Co., Shanghai, China). 

Mix 10 g of frozen pulp tissue from 20 jujubes with 30 mL pre-cooled (4 °C) 80% methanol, then grind the mixture into a homogenate under ice bath conditions. Centrifuge the homogenate at 4 °C and 12,000× *g* for 20 min, and collect the supernatant to determine ABTS radical scavenging ability at 734 nm following the method of Yu et al. [34]. The result was expressed as mmol Trolox equivalent (TE)·100 g^−1^.

### 2.9. Statistical Analysis

All measurements were taken in triplicate. SPSS 24.0 (IBM Corp., Armonk, NY, USA) was used to analyze the data following one-way analysis of variance and the results were presented as mean ± standard deviation (*n* = 3). Duncan’s test was used to determine the significant differences and *p* ≤ 0.05 was considered significant. Correlations among various indicators were analyzed using Pearson’s correlation test. Origin 2021 (OriginLab Corporation, Northampton, MA, USA) was used to generate plots.

## 3. Results and Discussion

### 3.1. Changes in the Respiration Rate, Decay Index, Weight Loss, TSS/TA and the Contents of TSS, TA, Total Soluble Sugar and Reducing Sugar

After harvest, Huping jujubes are still active and require the necessary energy and intermediate substances provided by respiratory metabolism for life activities [11,35]. However, respiration involves a series of redox reactions that consume sugars and organic acids as substrates and are closely related to storability and disease resistance [8]. Excessive respiration depletes organic matters, induces oxidative damage, decreases the disease resistance of fruits and leads to pathogenic microbial infection, thereby accelerating the ageing of fruits and vegetables and quality deterioration, such as decay, redness, dehydration and flavor loss [1,11,35]. Thus, regulating the respiratory metabolism is crucial for improving the postharvest quality of fruits.

**Figure 2:**
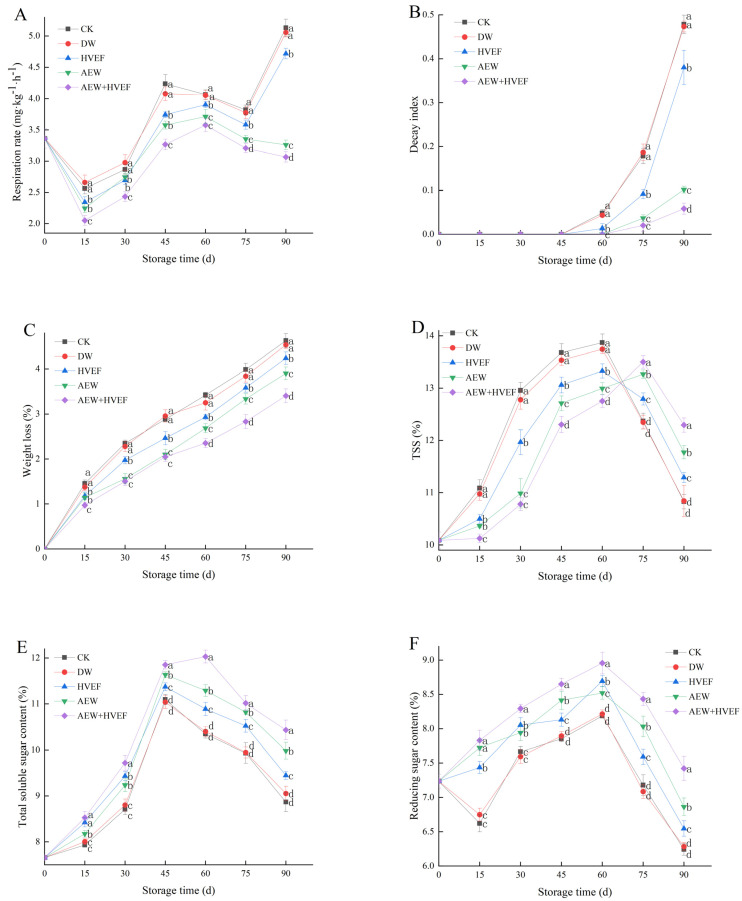

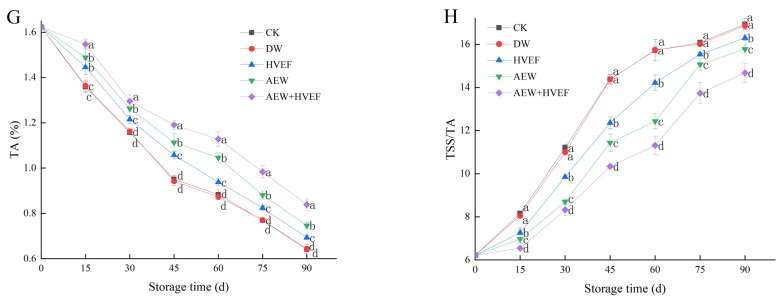
Effects of HVEF, AEW and AEW + HVEF treatments on respiration rate (**A**), decay index (**B**), weight loss (**C**), TSS (**D**), total soluble sugar content (**E**), reducing sugar content (**F**), TA (**G**) and TSS/TA (**H**) of Huping jujube during storage at 0 ± 1 °C for 90 days. The data presented are the mean values of three replicates; vertical bars represent the standard deviation of the mean values; values followed by different superscripts (a–d) are significantly different (*p* < 0.05) on the same sampling date.

From Figure 2A, there was a sudden drop in the respiration rate during the first 15 days due to a decrease in temperature. After that, the respiration rates of the CK and DW groups continuously increased and peaked on day 45, then decreased from day 45 to day 75, before finally rising sharply during the last 15 days. The last rising might be attributed to the serious infection of pathogens at the later stages of storage, as confirmed by Figure 2B. The HVEF group had a similar trend but peaked on day 60. However, the respiration rates of the AEW and AEW + HVEF groups increased after day 15, peaked on day 60 and then decreased. During storage, the AEW + HVEF group maintained the lowest respiration rate, followed by the AEW group, whereas the CK and DW groups showed no significant difference in respiration rates and retained the highest levels, followed by the HVEF group. Based on the overall trend, the HVEF, AEW and AEW + HVEF treatments not only effectively reduced respiration rates and peak values, but also delayed the appearance of the respiration peak by 15 days. It has been noted that the auxiliary group of oxidase related to respiratory metabolism of fruit contains iron, while HVEF can change the conformation of Fe^3+^-centered enzymes and prevent their activities, thereby blocking the electron transfer in the respiratory chain and inhibiting respiration [11]. Additionally, the proliferation and development of pathogens can exert a certain impact on the respiration rate of postharvest fruits [35], while AEW exhibits significant antimicrobial efficiency by altering the cell membrane structure of pathogens [25]; therefore, AEW treatment can inhibit the respiration rate.

The decay index is a quantitative index that reflects the severity of fruit infection by pathogens, which seriously affects the quality and commercial value [3,8]. In Figure 2B, the CK, DW and HVEF groups first showed apparent rot symptoms by day 60 with decay indices of 0.048, 0.043 and 0.013, respectively. However, the AEW and AEW + HVEF groups began to rot on day 75, which was 15 days later than the other three groups. On day 90, the decay indices of the CK, DW, HVEF, AEW and AEW + HVEF groups were 0.478, 0.473, 0.38, 0.102 and 0.058, respectively. Thus, it was suggested that HVEF, AEW and AEW + HVEF treatments notably curbed the increase in the decay index compared to the CK and DW groups. The lower decay index may be due to the remarkable bactericidal effects of HVEF and AEW [28,36]. AEW possesses lower pH, higher ORP and abundant active chlorine substances, such as ClO^−^ and HClO, which can induce the lysis of the external cell wall and membrane of the microorganisms, as well as directly destroy the nucleic acid and protein from within [25]. Meanwhile, HVEF can cause denaturation of bacterial proteins and deregulation of physiological metabolism by ionizing the air to generate ozone and negative air irons [11].

Weight loss is a crucial indicator of the freshness and edible quality of fruit, which generally increases during storage due to continuous respiration and transpiration [8]. In Figure 2C, the weight loss of all groups continuously increased. At each sampling time, the weight loss rates of the CK and DW groups showed no significant difference but were remarkably higher than those of other groups. The weight loss of the AEW + HVEF group was the lowest, followed by that of AEW. On day 90, the weight losses of the CK, DW, HVEF, AEW and AEW + HVEF groups were 4.6%, 4.5%, 4.2%, 3.9% and 3.4%, respectively. Previous studies reported that the weight loss occurred mainly due to nutrient consumption caused by respiration, transpiration and erosion or breakage of the epidermal tissue layer caused by reactive oxygen species (ROS) [18,36,37]. HVEF could reduce the stomatal opening on the epidermis [11] and alter the transmembrane potential of fruit and vegetable cells [18], thereby reducing the weight loss. Additionally, HVEF and AEW treatments can both inhibit the increase in the respiration intensity (Figure 2A) and decay index (Figure 2B) of Huping jujube, further decreasing the weight loss.

TSS, TA, total soluble sugar and reducing sugar are important nutrients and taste compounds, which can indirectly reflect the ripening and aging extent of fruit [4,8,35]. In Figure 2D, the TSS content initially increased followed by a subsequent decrease. The CK, DW and HVEF groups peaked on day 60, while AEW and AEW + HVEF groups peaked on day 75. At each sampling time before day 60, the TSS contents of all groups were, in declining order: CK(≈DW) > HVEF > AEW > AEW + HVEF (*p* < 0.05), while after day 60, the order changed to: AEW + HVEF > AEW > HVEF > CK(≈DW) (*p* < 0.05). Meanwhile, the total soluble sugar contents of all groups initially increased, reached their peaks on day 45 (except for the AEW + HVEF group with the peak on day 60) and then decreased (Figure 2E). After day 45, the total soluble sugar contents of all samples were, in decreasing order: AEW + HVEF > AEW > HVEF > CK(≈DW) (*p* < 0.05). On day 90, the total soluble sugar content of AEW + HVEF was 4.5% and 10.5% higher than that of AEW and HVEF groups, respectively. The reducing sugar contents of all samples (except for the CK and DW groups with a slight drop on day 15) showed a similar trend with the total soluble sugar (Figure 2F). On day 90, the reducing sugar content of AEW + HVEF was 8.2% and 13.5% higher than that of AEW and HVEF groups, respectively. Overall, the contents of TSS, total soluble sugar and reducing sugar exhibited a similar trend during storage, which is probably due to the hydrolysis of large molecular substances, such as starch, into soluble sugar as the fruit ripens. Subsequently, these soluble sugars were consumed and metabolized as respiratory substrates with the senescence of fruits [4,5,8].

The TA content is determined by the quantity of organic acids, which serve as important respiratory substrates and decrease with ripening [5]. In Figure 2G, the TA content continuously decreased during storage. Except for day 15, the TA contents of all groups decreased in the following order: AEW + HVEF > AEW > HVEF > CK(≈DW) (*p* < 0.05) at each sampling time. On day 90, the TA contents of CK, DW, HVEF, AEW and AEW + HVEF groups were 0.985%, 0.981%, 0.931%, 0.878% and 0.786% lower, respectively, than the initial content. The TSS/TA ratio served as an indicator of fruit acceptability, which reflected the overall taste and maturity of fruit. Generally, the higher the TSS/TA ratio, the lower the level of acceptability of fruit [5]. In this study, the changing trend of the TSS/TA ratio was opposite to that of the TA content (Figure 2H). On day 90, the TSS/TA ratio of the AEW + HVEF group was 9.9% and 7.0% lower than that of the HVEF and AEW groups, respectively.

To summarize, the HVEF, AEW and AEW + HVEF treatments could reduce the respiration rate and peak value, delay the appearance of the respiration peak, inhibit the increase in weight loss and decay index, and maintain lower TSS/TA and higher nutrient contents, such as those of TSS, total soluble sugar, reducing sugar and TA, thereby improving the sensory quality of fruit. Furthermore, the AEW + HVEF treatment exhibited superior preservation efficacy. It was reported that AEW (pH of 2.5 and ACC of 80 mg/L) could notably reduce the respiration rate, disease index and decay incidence, but maintain higher nutrient contents, including those of TSS, total soluble sugar and reducing sugar in harvested longan [28,29]. In addition, AEW (pH of 2.8, ORP of 1125 mV and ACC of 48 mg L^−1^) treated blueberries exhibited a lower incidence of decay and higher anthocyanin and total phenolics contents [26]. Additionally, HVEF could decrease the weight loss, respiratory rate and microbial loads of cherry tomato [18], persimmon [17] and tomato [19], as well as maintain higher TSS content of cranberry [20]. All of the abovementioned findings are in line with our results.

### 3.2. Changes in the Reddening Index, as Well as the Contents of Chlorophyll, Carotenoids and Flavonoids

**Figure 3:**
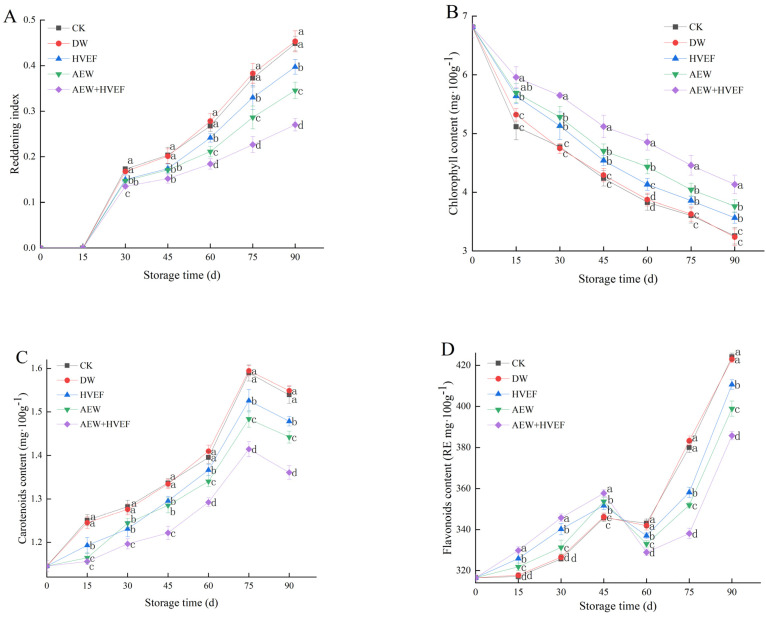
Effects of HVEF, AEW and AEW + HVEF treatments on reddening index (**A**), chlorophyll content (**B**), carotenoids content (**C**) and flavonoids content (**D**) of Huping jujube during storage at 0 ± 1 °C for 90 days. The data presented are the mean values of three replicates; vertical bars represent the standard deviation of the mean values; values followed by different superscripts (a–d) are significantly different (*p* < 0.05) on the same sampling date.

Color is the most intuitive reflection of fruit sensory quality, as well as the degree of oxidation and aging, which directly affects consumers’ willingness to purchase [3,16]. The reddening index, an important physicochemical property, is used to reflect the extent of color transformation [6]. In Figure 3A, the reddening indices of all groups began to continuously increase after day 15. At each sampling time after day 45, the AEW + HVEF group maintained the lowest levels of reddening index, followed by the AEW group. However, the reddening indices between the CK and DW groups showed no significant difference but were higher than those of other groups. On day 90, the reddening index of the AEW + HVEF group was 7.6% and 12.7% lower than that of the AEW and HVEF groups, respectively, while that of the AEW group was 10.7% lower than that of the DW group, and that of the HVEF group was 5.1% lower than that of the CK group.

The pericarp color transformation from green to yellow and finally to red is mainly due to the degradation of chlorophyll, as well as the accumulation of total carotenoids and flavonoids [3,4,5,9]. Chlorophyll is a major photosynthetic pigment and the higher the chlorophyll content, the lower the maturity of fruit [3,10]. In Figure 3B, the chlorophyll content continuously decreased during storage. Throughout the entire storage, the fruits treated with AEW + HVEF maintained the highest chlorophyll contents, followed by the AEW and HVEF groups, which showed no remarkable difference (except for day 60). Additionally, there was also no obvious difference between the CK and DW groups, which showed the lowest chlorophyll contents. On day 90, the chlorophyll contents of the CK, DW, HVEF, AEW and AEW + HVEF groups were 52.2%, 52.6%, 47.7%, 44.8% and 39.4% lower than the initial content, respectively. Chlorophyll degradation is closely related to the disintegration of thylakoid membranes, which contain abundant photosynthetic pigments and electron transport chains. The electric field can not only preserve the thylakoid membrane by decreasing cellular damage and free radical generation, but also inhibit chlorophyllase activity and slow down cellular metabolism by reducing the membrane potential difference and the extent of oxidative phosphorylation, thereby leading to slower degradation of chlorophyll [11]. 

Carotenoids, appearing only after chlorophyll degradation, are naturally synthesized plant pigments that can stimulate the yellow transformation of the pericarp [10,28]. In Figure 3C, the carotenoid contents of all samples increased until day 75, then decreased. The carotenoid contents of the CK and DW groups showed no notable difference, but were obviously higher than those of the other three groups throughout storage. After day 60, the carotenoid contents of the HVEF group were higher than those of the AEW group, followed by those of the AEW + HVEF group (*p* < 0.05). On day 90, the carotenoid content of the AEW + HVEF group was 5.6% and 7.9% lower than that of the AEW and HVEF group, while that of the AEW group was 6.9% lower than that of the DW group and that of the HVEF group was 4.0% lower than that of the CK group.

Flavonoids, which contain a variety of different derivatives such as anthocyanin, catechin and naringenin, present a prominent influence during the reddening of fruit peel [3,10]. In Figure 3D, the flavonoid contents increased before 45 days, then decreased on day 60, finally sharply rosing until day 90. It was reported that the increase in flavonoid content before 45 days was associated with the antioxidant activity of fruit, while the decrease on day 60 and subsequent sharp rise were related to the fruit reddening [10]. During storage, the flavonoid contents of the AEW + HVEF group maintained the highest levels from day 0 to 45, but showed the lowest levels from day 60 to 90, while those of the CK and DW groups were reversed. Except for day 45, the flavonoid contents of the HVEF group were memorably higher than those of the AEW group (*p* < 0.05) during storage. On day 90, the flavonoid content of the AEW + HVEF group was 3.3% and 6.1% lower than that of the AEW and HVEF groups, respectively, while that of the AEW was 5.7% lower than that of the DW group and that of the HVEF group was 3.2% lower than that of the CK group.

In summary, the AEW + HVEF treatment could better delay the degradation of chlorophyll and accumulation of carotenoids and flavonoids than the treatments of HVEF and AEW alone, which could explain why the reddening index was lowest in the AEW + HVEF group.

### 3.3. Changes in Color Characteristics

**Figure 4:**
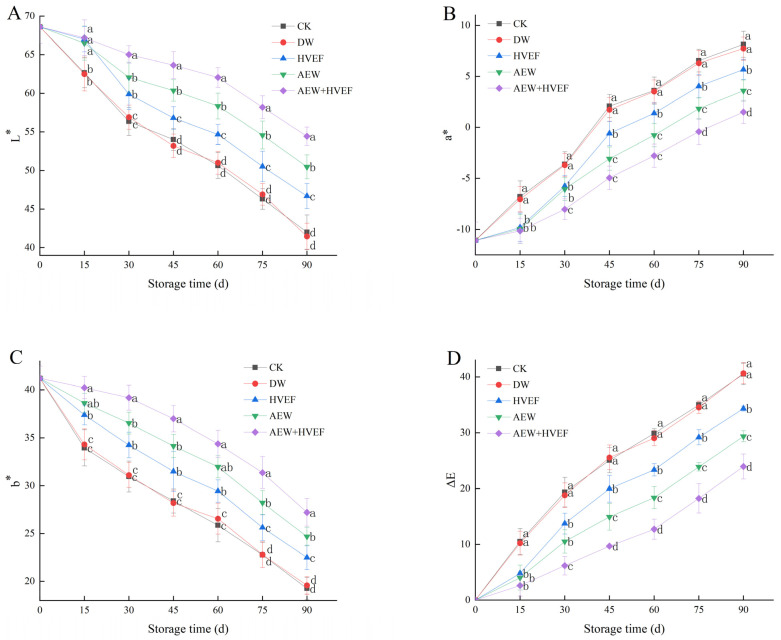
Effects of HVEF, AEW and AEW + HVEF treatments on L* (**A**), a* (**B**), b* (**C**) and ΔE (**D**) of Huping jujube during storage at 0 ± 1 °C for 90 days. The data presented are the mean values of three replicates; vertical bars represent the standard deviation of the mean values; values followed by different superscripts (a–d) are significantly different (*p* < 0.05) on the same sampling date.

Apart from the reddening index and the pigment contents, L*, a*, b* and ΔE values are also normally used to evaluate the apparent color characteristics of fruits [30,35]. The L* value represents the brightness of the color, while the a* and b* values express the color changes from green to red and blue to yellow, respectively [28]. In Figure 4, the L*and b* values decreased continuously, while the a* and ΔE values increased during storage. The CK and DW groups showed little difference in L*, a*, b* and ΔE values at each sampling period. However, the L* and b* values of the two groups were significantly lower than those of the other three groups, while the a* and ΔE values were notably higher. By contrast, the AEW + HVEF group exhibited remarkably higher L* and b* values than other groups during storage, while showing observably lower a* and ΔE values. Additionally, the L* and b* values of the AEW group were significantly higher than those of the HVEF group after 30 and 60 days, respectively. Meanwhile, the a* and ΔE values of the AEW group were memorably lower than those of the HVEF group after 30 days. On day 90, the L* values of CK, DW, HVEF, AEW and AEW + HVEF groups decreased by 38.8%, 39.6%, 32.0%, 26.5% and 20.7% from the initial value, respectively, while the b* values of CK, DW, HVEF, AEW and AEW + HVEF groups were 53.3%, 52.5%, 45.4%, 40.11% and 34.0% lower than the initial value, respectively. However, the a* value of the AEW + HVEF group was 53.4% and 69.4% lower than that of the AEW and HVEF groups on day 90, respectively. Similarly, the ΔE value of the AEW + HVEF group was 17.5% and 29.1% lower than that of the AEW and HVEF groups, respectively. 

Previous studies showed that AEW (pH of 2.5 and ACC of 80 mg/L) could retard the degradation of pericarp pigments including carotenoids, chlorophyll and flavonoids, and suppress the decrease in L*, a* and b* values of longan, thereby keeping a higher rate of commercial acceptability [28,29]. Similarly, SAEW (pH of 6.0, ORP of 1340 mV and ACC of 80 mg/L) could delay the increase in a*, b* and carotenoids content, as well as the decrease in L*, hue angle and chlorophyll content, thereby reducing the peel browning and yellowing indices of carambola and retaining better apparent color [29]. SAEW (pH of 5.95 ± 0.11, ORP of 922 ± 9 mV and ACC of 29.8 ± 0.5 mg L^−1^) significantly retarded the loss of green color and skin lightness in ‘Jiancui’ jujubes [33]. Meanwhile, HVEF (−2 kV cm^−1^) could delay the decline of chlorophyll content in tomato [19]. HVEF (150 kV/m) maintained a higher L* value and lower ΔE of cherry tomato [18]. In addition, mushrooms treated with HVEF showed better skin color by maintaining a higher whiteness index and L*, as well as lower a* and b* [38]. Additionally, HVEF could effectively preserve the color of fresh-cut broccoli [16], button mushroom [15] and cranberry [20]. 

Our results showed that the HVEF, AEW and AEW + HVEF treatments could slow the rise in a* and ΔE values and the drop in L* and b* values, thereby improving the appearance quality, which was in agreement with previous findings. Moreover, the AEW + HVEF treatment presented better visual appearance of jujube than either treatment alone. 

### 3.4. Changes in GR Activity and ABTS Radical Scavenging Ability

**Figure 5:**
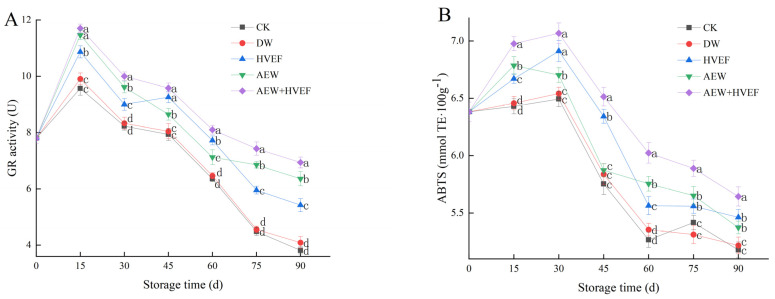
Effects of HVEF, AEW and AEW + HVEF treatments on the GR activity (**A**) and ABTS radical scavenging ability (**B**) of Huping jujube during storage at 0 ± 1 °C for 90 days. The data presented are the mean values of three replicates; vertical bars represent the standard deviation of the mean values; values followed by different superscripts (a–d) are significantly different (*p* < 0.05) on the same sampling date.

Tissue aging and quality deterioration of fruits are closely related with the excessive accumulation of ROS [11]. GR provides reductive power for eliminating the excessive ROS by reducing oxidized glutathione to reduced glutathione, thereby protecting fruits from oxidative damage and delaying senescence [4]. In Figure 5A, the GR activities of all groups increased before day 15, then continuously declined. The GR activities in the CK and DW groups showed little difference but retained the lowest values at each sampling time, while those in the AEW + HVEF group were the highest during storage. However, except for days 45 and 60, the GR activities in the AEW group were higher than those in the HVEF group during storage (*p* < 0.05). On day 90, the GR activity was 9.1% and 18.4% higher in the AEW + HVEF group than in the AEW and HVEF groups, respectively. 

ABTS+-scavenging ability is a crucial parameter for evaluating the antioxidant capacity and determining the preservation quality of fruits [34,39]. In Figure 5B, except for a slight rise in the CK group on day 75, the ABTS radical scavenging abilities of all samples increased initially, peaked on day 30 and then decreased. The ABTS+-scavenging abilities of the CK and DW groups exhibited no significant difference but were notably lower than those of other groups at each sampling time. In contrast, the AEW + HVEF group showed significantly higher ABTS+-scavenging ability than other groups. The ABTS+-scavenging abilities of the HVEF group were higher on days 30 and 45, and lower on days 15 and 60, than those of the AEW group, but were similar to those of the AEW group during the last 60 days. On day 90, the ABTS+-scavenging ability was 5.0% and 3.3% higher in the AEW + HVEF group than in the AEW and HVEF groups, respectively, while it was 2.9% higher in the AEW group than in the DW group and 5.4% higher in the HVEF group than in the CK group.

Overall, AEW + HVEF treatment could better improve the quality of Huping jujube during storage than a single application by enhancing GR activity and ABTS scavenging ability. A previous study reported that AEW (ACC of 60 mg/L, pH of 2.2 and ORP of 1177 ± 5 mV) can improve the postharvest quality of ‘Lingwu Long’ jujube by increasing the GR activity, phenylpropanoid metabolism and cell wall metabolism [27]. Meanwhile, direct-electric-current treatment could enhance the ABTS+-scavenging ability of tomato [40]. In addition, the GR activity and ABTS+-scavenging ability of jujube could also be enhanced by the incorporation of 1-methylcyclopropene(1-MCP) and salicylic acid [4], ultraviolet [3], and a composite of 1-MCP, chitosan and natamycin [34]. The above findings showed results that were similar to those of our work.

### 3.5. Correlation Analysis of the Effect of AEW + HVEF Treatment on the Physiological Metabolism in Huping Jujube

Based on our results, the fruits treated with AEW + HVEF showed the best storage quality. To better investigate the relationships between different indices and their impacts on storage quality, Pearson’s correlation analysis was conducted on different characteristics of AEW + HVEF treatment mentioned in this study during storage.

**Figure 6:**
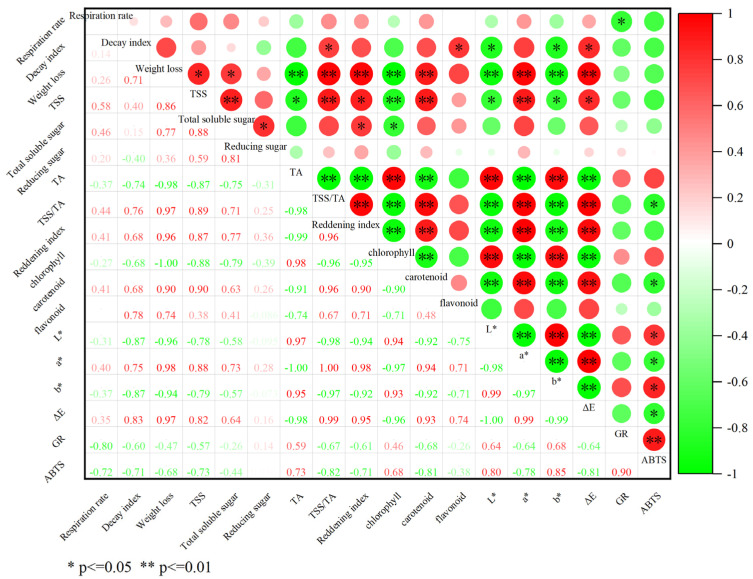
Pearson’s correlation matrix of the respiration rate, nutrients and taste compounds, appearance color and antioxidant capacity of Huping jujube treated by AEW + HVEF for 90 days. The correlation coefficients are proportional to numerical size and color intensity. Red color represents a positive correlation and green color represents a negative correlation. Correlation levels significant at * *p* < 0.05 and ** *p* < 0.01, respectively.

In Figure 6, the decay index correlated positively with the TSS/TA, flavonoids and ΔE but negatively with L* and b* (*p* < 0.05). Furthermore, the reddening index correlated positively and highly significantly with carotenoids, a*, ΔE, weight loss and TSS/TA (*p* < 0.01) but negatively with chlorophyll, L*, b* and TA (*p* < 0.01). It was indicated that the quality deterioration of fruit was accompanied by dehydration, changes in pigments and chromaticity values, and degradation of nutrients and taste compounds. Additionally, GR activity was correlated strongly and positively with the ABTS+-scavenging ability (*p* < 0.01), but negatively with respiration rate (*p* < 0.05), which suggested that GR played a key role in removing free radicals, improving antioxidant capacity, and prolonging the storage life of Huping jujube. The above results were consistent with the findings of previous studies [8,35]. 

## 4. Conclusions 

In summary, the AEW, HVEF and AEW + HVEF treatments could better preserve the storage quality of Huping jujube during cold storage than the CK and DW treatments. Firstly, the three treatments not only reduced the respiration rates and peak values, but also delayed the appearance of the respiration peak by 15 days compared to the CK and DW groups. Next, the treated fruits could maintain lower levels of weight loss, decay index and TSS/TA, as well as higher levels of nutrients and taste compounds, including TSS, total soluble sugar, reducing sugar and TA. In addition, the pericarp color of the treated fruits exhibited lower reddening index, a* and ΔE values, as well as higher L* and b* values by retarding the degradation of chlorophyll and accumulation of carotenoids and flavonoids. Finally, the three treatments could enhance the GR activity and ABTS+-scavenging ability. However, the fruits treated with AEW + HVEF exhibited better storage quality than those treated with either AEW or HVEF alone. Correlation analysis indicated that the storage quality of jujubes was closely related with respiration, dehydration, color, nutrients and taste compounds, as well as antioxidant capacity. Although AEW + HVEF treatment has been shown to improve the storage quality of Huping jujube, the molecular mechanism has not yet been revealed. Therefore, we will conduct metabolomic, transcriptomic and microbiome studies to elucidate the bactericidal and preservation mechanisms of AEW + HVEF from a broader metabolic, genetic and microbial perspective.

## Data Availability

Data is contained within the article.
